# Supplementary material for: The interactome of intact mitochondria by cross-linking mass spectrometry provides evidence for coexisting respiratory supercomplexes
Source: Mol Cell Proteomics. 2017 Dec 8;17(2):216–32. doi: 10.1074/mcp.RA117.000470 (PMC5795388; doi:10.1074/mcp.RA117.000470)
Supplement: Supplemental Data [file supp_RA117.000470_133922_0_supp_23978_fzffwf.zip › spectra_annotation/mito_spectra_annotation/46-1-1-1-5-1.pdf]

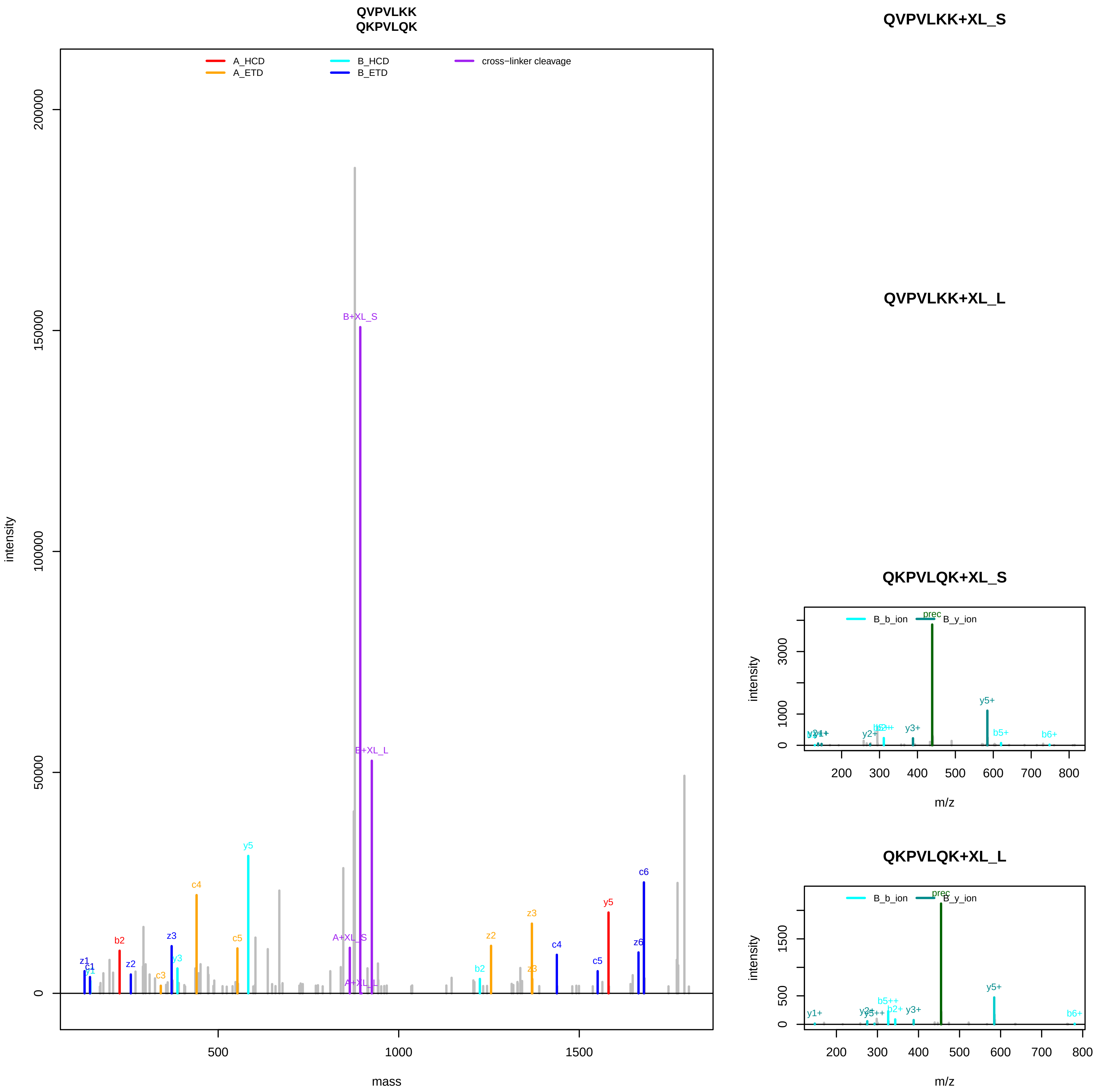
QVPVLKK
QKPVLQK
A_HCD
A_ETD
B_HCD
B_ETD
cross−linker cleavage
intensity
mass
0
50000
100000
150000
200000
500
1000
1500
z1
c1
y1
b2
z2
c3
z3
y3
c4
c5
y5
A+XL_S
A+XL_L
B+XL_S
B+XL_L
b2
z2
z3
c4
c5
y5
z6
c6
QVPVLKK+XL_S
QVPVLKK+XL_L
QKPVLQK+XL_S
B_b_ion
B_y_ion
prec
y5+
b5+
b6+
y2+
y3+
m/z
QKPVLQK+XL_L
y1+
b5++
b2+
200
300
400
500
600
700
800
